# Roles of Insect Oenocytes in Physiology and Their Relevance to Human Metabolic Diseases

**DOI:** 10.3389/finsc.2022.859847

**Published:** 2022-03-17

**Authors:** Kerui Huang, Ying Liu, Norbert Perrimon

**Affiliations:** ^1^Department of Genetics, Harvard Medical School, Blavatnik Institute, Boston, MA, United States; ^2^Harvard Medical School, Howard Hughes Medical Institute, Boston, MA, United States

**Keywords:** oenocytes, *Drosophila*, lipid metabolism, tissue communication, hepatocyte, adipocyte, fat body cells, hydrocarbon

## Abstract

Oenocytes are large secretory cells present in the abdomen of insects known to synthesize very-long-chain fatty acids to produce hydrocarbons and pheromones that mediate courtship behavior in adult flies. In recent years, oenocytes have been implicated in the regulation of energy metabolism. These hepatocyte-like cells accumulate lipid droplets under starvation and can non-autonomously regulate tracheal waterproofing and adipocyte lipid composition. Here, we summarize evidence, mostly from *Drosophila*, establishing that oenocytes perform liver-like functions. We also compare the functional differences in oenocytes and the fat body, another lipid storage tissue which also performs liver-like functions. Lastly, we examine signaling pathways that regulate oenocyte metabolism derived from other metabolic tissues, as well as oenocyte-derived signals that regulate energy homeostasis.

## Introduction

Regulating energy utilization and storage is central to animal physiology and adaptation to environmental challenges. Under conditions of nutrition surplus, glucose is converted to fatty acids, which are then synthesized into triglycerides (TGs) and stored as lipid droplets. Excessive lipid stores can be detrimental and have been associated with various metabolic diseases, such as cardiovascular diseases (CVDs), non-alcoholic fatty liver disease (NAFLD), obesity and insulin resistance, making understanding lipid metabolism of great importance to human health.

The liver is the major detoxifying organ of the body and plays a central role in regulating the metabolism of carbohydrates, proteins and lipids. Moreover, the liver is the major site for glycogen storage and very low-density lipoprotein (VLDL) secretion (1, 2). During starvation, adipocytes undergo lipolysis to produce free fatty acids (FFAs). FFAs are processed by hepatic oxidation to generate ketone bodies in the liver which are then used as fuels for other tissues. If mobilization of FFAs exceeds the rate of lipid oxidation, re-esterification of surplus FFAs to TGs occurs in the liver, leading to an increase in intrahepatic TG content, i.e., steatosis. NAFLD, a common manifestation of the metabolic syndrome, is characterized by steatosis in the absence of starvation. Nonalcoholic hepatic steatosis is present in approximately 25% of the adult population worldwide, and NAFLD is the most common liver disease in Western societies. Thus, understanding how hepatic diseases regulate cellular processes in peripheral organs and how other organs contribute to steatosis is of interest to human metabolic diseases.

Major metabolic and endocrine pathways are conserved in *Drosophila*, making this model organism well suited to dissect the cellular and molecular mechanisms underlying physiology (3–5). The fly fat body is equivalent to the vertebrate white adipose tissue (WAT), which stores excess fat as TGs. In addition, fly oenocytes, which are similar to hepatocyte cells, are important for mobilizing stored lipids from the fly fat body (6). Like mammals, flies convert excess carbohydrates into TGs through *de novo* lipogenesis (7, 8). In addition, excess carbohydrates and amino acids can also be processed into UDP-glucose, which fuels glycogen synthesis (9). Regulation of energy storage in flies involves several signaling pathways, including insulin/insulin-like growth factor (IGF) signaling, which is similar to the insulin signaling in mammals (10). However, unlike mammals, there are eight different *Drosophila* insulin-like peptides (dILPs). Most of these modulate the IGF pathway through a single insulin receptor, InR (10, 11). Under nutrient-deprivation or energy demanding conditions, lipids are released from the fat body through increased lipolysis (12), and are further processed in oenocytes (6, 13). Signaling that regulates catabolism of lipids and carbohydrates include adipokinetic hormone (Akh), which is similar to glucagon in mammals and ecdysone, which antagonizes insulin signaling (14, 15).

In this review, we explore the potential of *Drosophila* oenocytes as a model for hepatic diseases. We summarize the different roles of oenocytes and the fat body in regulating carbohydrate and lipid metabolism under normal or starved conditions. We also discuss the intricate interplay of oenocytes with other tissues, including the fat body and muscles, in shaping organismal lipid storage.

## Oenocytes as the Lipid Metabolizing Center

Oenocytes were originally described as wax-producing cells because histological stains and organic extractions suggested that they contain wax particles or other lipids (16). These unusual cells contain abundant smooth endoplasmic reticulum (ER), which synthesizes lipids, phospholipids, steroids and metabolizes carbohydrates (17). In addition, oenocytes are also highly enriched in peroxisomes (18), the major sites for metabolism of reactive oxidative species (ROS) and β-oxidation of very-long-chain fatty acids. Both smooth ER and peroxisomes are highly enriched in mammalian hepatocytes, highlighting the functional similarities between oenocytes and liver cells in lipid metabolism.

**Figure 1:**
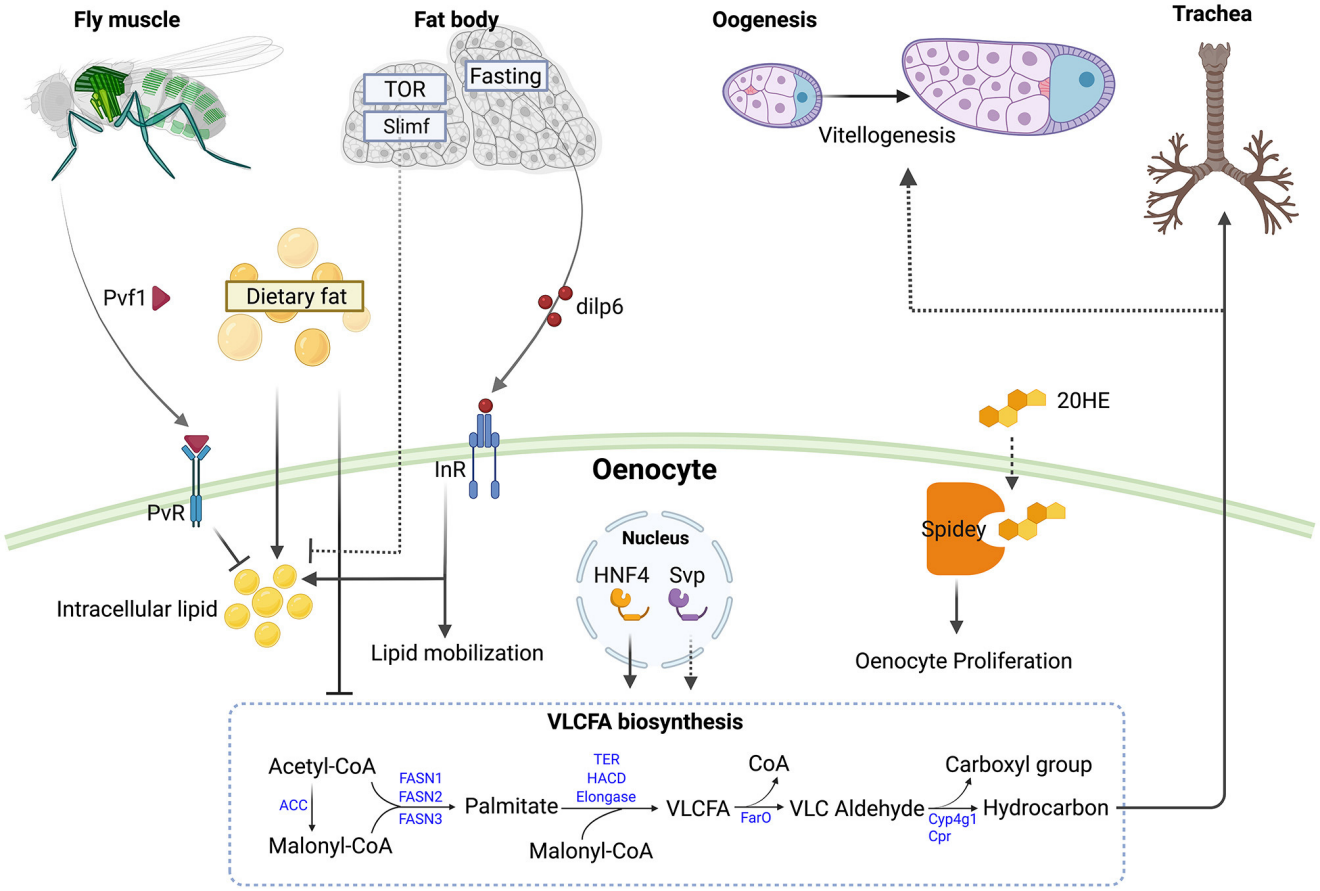
Roles of oenocytes in modulating metabolism and responding to nutritional signal. Muscle derived PDGF- and VEGF-related factor 1 (Pvf1) signals to PDGF- and VEGF-receptor related (PvR) in oenocytes to inhibit lipid accumulation and lipogenesis in adult flies (27). Elevated levels of circulating fats can promote oenocyte steatosis and inhibit very long chain fatty acid biosynthesis (28). Knockdown of *target of rapamycin* (*TOR*) or *slimfast* (*slif*) in the larval fat body increase steatosis in oenocytes (6). During fasting, the adult fat body produces Insulin-like peptide 6 (Ilp6) which activates the Insulin-like receptor (InR) in oenocytes to promote lipid accumulation and mobilization (13). The nuclear receptors Hepatocyte nuclear factor 4 (Hnf4) and seven up (svp) in adult oenocytes regulate very long chain fatty acid (VLCFA) biosynthesis (26, 29). Knockdown of *svp* in oenocytes impairs vitellogenesis in ovaries, possibly by reducing hydrocarbon production (30). Spidey in oenocytes can metabolize 20-hydroxyecdysone (20HE) and promotes oenocyte proliferation (26). Larval oenocyte-derived hydrocarbon can waterproof the trachea (31). The VLCFA biosynthesis pathway is depicted with *Drosophila* genes (in blue) (29). Abbreviations in the pathway: ACC, Acetyl-CoA carboxylase; FASN1-3, Fatty acid synthase 1-3; TER, HACD and Elongase are each encoded by multiple genes; FarO, Fatty acyl-CoA reductase in oenocytes; Cyp4g1, cytochrome P450 4g1; Cpr, Cytochrome P450 reductase. Figure is created with BioRender.com.

Oenocytes in different insects have been shown to change with the molting cycle (16), prompting investigation of the role of oenocytes in production of the insect hormone ecdysone. The active form of ecdysone, 20-hydroxyecdysone (20E), which is the primary molting hormone, regulates a variety of physiological processes, including metamorphosis, immune response, and reproduction (19–21). In larvae, ecdysone is mainly synthesized in the prothoracic gland from its cholesterol precursor via a set of cytochromes P450 proteins encoded by the “Halloween genes” (22). In adults, ecdysone is mainly but not exclusively synthesized in ovary (23). Ecdysone is converted to 20HE in peripheral tissues, such as the fat body, Malpighian tubules, and midgut. These tissues express *shade (shd)*, which encodes an E-20-monooxygenase that mediates the hydroxylation of ecdysone at carbon 20 (24). Interestingly, two of the ecdysone biosynthesis genes, *Phantom* and *Shadow*, are highly expressed in adult oenocytes (25), suggesting that oenocytes participate in ecdysone biosynthesis. Further, manipulation of *spidey*, which encodes a steroid dehydrogenase, regulates ecdysone metabolite levels (26). Silencing or overexpression of *spidey* during embryonic development results in pupal lethality, similar to what is observed for mutations in ecdysone signaling pathway genes (26). In addition, oenocyte-specific knockdown of *spidey* in larvae results in accelerated oxidation of 20HE, while overall 20HE levels remain unchanged. Finally, overexpression of *spidey* in oenocytes leads to dramatic reduction of 20HE and its catabolic metabolites (26) (Figure 1). In further support of the role of oenocytes in ecdysone biosynthesis, isolated oenocyte-fat body complexes (OEFC) from adult males of the cricket *Gryllus bimaculatus* have been found to secrete ecdysteroids (32). Moreover, ecdysone has been linked to lipid metabolism in various tissues (33–35), suggesting that oenocytes regulate lipid metabolism by modulating ecdysone levels. It remains to be elucidated whether oenoytes contribute to ecdysone synthesis during adult or larval stages, a question that could be addressed using genetic ablation of the oenocytes. Furthermore, the functional significance of ecdysone in oenocytes physiology and metabolism is of interest.

In line with the central role in lipid metabolism, oenocytes are also the major sites for VLCFAs synthesis, which are constituents of cellular lipids such as sphingolipids and cuticular hydrocarbons. Oenocyte ablation during larval stages using the *GAL4/UAS-reaper* system leads to compromised tracheal air-filling and the larvae exhibit hypoxia-induced phenotypes in larvae. Strikingly, oenocyte ablation or knockdown in larvae of genes encoding VLCFA metabolizing enzymes (*ACC, KAR, elongase*) result in severe tracheal defects, with the tracheal tubes filled with an aqueous solution (31). Interestingly, the spiracle ducts, which are connected to the trachea and correspond to the respiratory openings found on the thorax and abdomen of larvae, are no longer filled with Oil Red O-staining in oenocyte-ablated or *Acc* mutant larvae (31), suggesting that larval oenocytes might provide VLCFAs that coat the trachea. Alternatively, VLCFAs, which are synthesized from oenocytes, can signal non-autonomously to control lipid metabolism in spiracles, which have been proposed to obtain waterproofing lipids from specialized spiracular gland cells (Figure 1) (16).

In adult flies, cuticular hydrocarbons have been found to provide protection from desiccation and to act as pheromones for sexual communication and modulate longevity (16, 36, 37). Previous studies have suggested that cytoplasmic projections from the oenocytes contact the epidermis and that these cell-to-cell contacts facilitate lipid or lipoprotein transfer from oenocytes to the epidermis (38–42). Adult male and female flies in which oenocytes have been ablated using the *GAL4/UAS* system, show reduced levels of most of the cuticular hydrocarbons (36). Oenocyte-specific knockdown of genes regulating hydrocarbon production, such as *CYP4g1*, leads to reduced desiccation resistance (43). Further, mutations in the nuclear receptor Hnf4, which is strongly expressed in oenocytes (6), show reduced expression of genes involved in VLCFA biosynthesis, including *KAR, CYP4g1, Cpr* and genes encoding elongases; reduced levels of hydrocarbons; and decreased dry starvation resistance (Figure 1) (29). Interestingly, mouse hepatocytes isolated from mice with a mutation in *HNF4*α, the mammalian ortholog of *Hnf4*, also show markedly decreased expression of genes that encode elongases, i.e., *Elovl3* or *Elovl5*. Mice mutant for *Elovl1* and *Elovl4* die shortly after birth from acute dehydration and loss of epidermal hydrophobic barrier function (44, 45), suggesting that the regulation of VLCFA biosynthesis by HNF4 is evolutionarily conserved.

## *Drosophila* Oenocytes as an Emerging Model for Liver Function

Studies of oenocytes focused on their role in hydrocarbon synthesis until Gutierrez et al. showed that larval oenocytes can store and process lipids under starvation, a function analogous to what occurs in the mammalian liver. Under fed conditions, *Drosophila* larvae store lipids in the midgut epithelial cells and the fat body (6, 46). However, after 14 h of fasting, lipid droplets can no longer be detected in the fat body and the midgut, but intense Oil Red O staining persists in the oenocytes (6). Observation of this starvation induced steatosis suggested that larval oenocytes have hepatocyte-like functions. Further, although the progenitors of larval and adult oenocytes are different (16), Chatterjee et al. showed that like larval oenocytes, adult oenocytes also exhibits starvation-induced steatosis and that this process is mediated by fat body secreted *Drosophila* insulin-like peptide 6 (dILP6) (13). Under acute fasting response, adult female flies accumulate lipid droplets in the oenocytes, although the level of this steatosis is mild and more heterogenous as compared to what is observed in larvae. In starvation conditions, several genes with catabolic and gluconeogenic functions in hepatocytes are also highly induced in oenocytes, including *amylase proximal (amy-P)* and *phosphoenolpyruvate carboxykinase (pepck)*, which catalyzes the rate-limiting step in gluconeogenesis (13).

In addition to gluconeogenic genes, which are induced in oenocytes in response to starvation response, oenocytes are also enriched with genes involved in ketogenesis, long-chain fatty acid metabolism, and peroxisomal function, all of which are also enriched in the mammalian liver. Huang et al. performed oenocyte-specific translatomic profiling using the RiboTag sequencing approach to explore this. By comparing oenocyte RiboTag data with previously published fly whole body transcriptome datasets, these authors identified genes enriched in adult oenocytes (25). Further, by comparing these genes to genes enriched in mammalian liver, Huang et al. identified several commonly enriched genes. One of them is *HMG-CoA synthase* (*Hmgs* in flies and *HMGCS1/2* in humans), which encodes the key enzyme involved in ketogenesis and cholesterol biosynthesis. Others include *HMG-CoA lyase (CG10399* in flies and *HMGCL* in humans) and *D-*β*-hydroxybutyrate dehydrogenase (shroud* in flies and *BDH1* in humans), which encode key enzymes involved in ketogenesis. These observations suggest that oenocytes may be the primary site for ketogenesis in flies. Our current understanding of ketone body metabolism in insects is limited to locusts. Bailey et al. showed that the hemolymph of the locust *Schistocerca gregaria* contains appreciable levels of acetoacetate and at least a small amount of 3-hydroxybutyrate (47). The levels of acetoacetate, but not of 3-hydroxybutyrate, increase during starvation and flight. This is different from mammals, in which 3-hydroxybutyrate is the dominating ketone body. It seems possible that *Drosophila* oenocytes produce alternative ketone bodies, including acetoacetate, under flight or starved condition.

Genes involved in the synthesis of VLCFAs and microsomal fatty acid elongation are also highly enriched in oenocytes and the liver (25). These include very-long-chain 3-ketoacyl-coA synthase (*CG18609* in flies and *ELOVL2* in humans), which catalyzes the first step of VLCFA synthesis in smooth ER (25), and several key genes involved in the production of cuticular hydrocarbons, such as *Cyp4g1, Cpr, FarO* (16, 28, 29, 41). Notably, the role of the mammalian liver in synthesizing hydrocarbons is unclear, although it has been shown that VLCFA genes (*ELOVL2* and *ELOVL6*) are enriched in this organ. Fibroblast growth factor 21 (*branchless* in fly and *FGF21* in human), is also a key hormonal factor that is enriched in both oenocytes and the liver (25). *FGF21* is an important metabolic regulator that has anti-diabetic properties in humans (48). Several studies have shown that FGF21 stimulates fatty acids oxidation, ketone body production and inhibits lipogenesis (49, 50). Interestingly, exogenously provided FGF21 increases longevity and stress tolerance in female silkworms (*Bombyx mori)*, possibly through activated *AMPK, FoxO*, and sirtuins (51), suggesting that insects can be used as animal models for evaluating the pharmaceutical effects of FGF21. The role of *FGF21* in fly oenocytes remains to be characterized and may provide an excellent model to further decipher the role of FGF21 in oenocyte/liver function.

## Comparison of the Roles of Oenocytes and the Fat Body With Liver Functions

In addition to oenocytes, the fat body has also been regarded as a liver-like tissue in *Drosophila*, given its roles in nutrient sensing, glycogen storage, detoxification, the immune response, and lipid storage (3). Interestingly, comparison of oenocyte- and fat body-enriched genes revealed that there was very little overlap between these two tissues (25), suggesting that they perform distinct biological functions. Indeed, Gene Ontology (GO) analysis revealed an enrichment of genes involved in carboxylic acid and amino acid metabolism in the fat body, whereas oenocytes showed an enrichment for genes involved in fatty acid elongation, biosynthesis, xenobiotic metabolism and peroxisomal function (25). See a summary of comparison of liver function with the fat body and the oenocyte in Table 1.

**Table 1:** Summary of liver function compared with fat body and oenocytes.

**Liver**	**Fat body**	**Oenocyte**	**References**
Lipoprotein production	Yes	?	(51)
Glycogen metabolism	Yes	?	(52)
Starvation induced steatosis	No	Yes	(6, 13)
Ketogenesis	?	?	(25, 46)
Innate immune response			(25)
Detoxification of chemicals	Yes	Yes	(25, 53–56)
Amino acid metabolism	Yes	?	(54–57)
Clotting regulation	?	?	
Bile acid production	?	?	
Cholesterol metabolism	?	?	

In mammals, the liver and gut are the two primary organs for the secretion of lipoproteins, which deliver lipids and sterols to peripheral tissues. Apolipoprotein B (ApoB) is the primary apolipoprotein that scaffolds chylomicrons and very low-density lipoproteins (VLDL), which are secreted by the gut and liver, respectively. In *Drosophila* and other insects, lipophorins (Lpp) are the major lipoproteins similar to apoB-containing lipoproteins in mammals (58). Lpp scaffolding apolipoproteins are the highly conserved apolipophorins (apoLpp) (59). In addition, the two *Drosophila* lipoprotein receptors (LpR1 and LpR2), homologous to LDL receptors in mammals (60, 61), can promote Lpp uptake. A BLAST search against human apoB yields four *Drosophila* genes: *apolipophorin* (*apolpp), microsomal triacylglycerol transfer protein (Mtp), apolipoprotein lipid transfer particle (Apoltp)* and *crossveinless d (cv-d)* (58). *Drosophila* larvae hemolymph contains three circulating lipoproteins: Lpp, LTP, and Cv-d. Among them, Lpp is the major lipid carrier as more than 95% of hemolymph lipids co-fractionate with it (58). Interestingly, the larval fat body is the primary tissue that secretes Lpp, as fat body-specific *Lpp* knockdown strongly reduces the level of circulating Lpp and LTP in the hemolymph. Based on this, the fat body performs liver-like function regulating lipid transport through secreted lipoproteins. It remains to be determined whether and how oenocytes regulate lipoprotein circulation in the hemolymph, as four apoB genes are also expressed in oenocytes. In addition, oenocyte-specific over-expression of the lipogenic genes fatty acid synthase 1 and 3 (*fasn1* and *fasn3)* can increase lipid droplet size in the fat body, suggesting that lipids generated from oenocytes are transported to the fat body, possibly through lipoproteins (27).

The key liver-like characteristic of the fat body is glycogen storage and utilization. In humans, glycogen is primarily stored in the liver (~100 g) and in skeletal muscles (~500 g). However, human muscles do not show major decreases in glycogen during fasting (52, 62), and only liver stored glycogen contributes to the release of glucose into the blood, specifically during fasting. Thus, the liver is viewed as a “glucostat” that maintains circulating sugar levels (63). Net hepatic glycogen synthesis is one of the major direct effects of insulin on hepatocytes and an important mechanism for suppression of hepatic glucose production (64). During fasting, the pancreas secretes glucagon to initiate a cascade of kinase activity that leads to release of glucose from stored glycogen via glycogenolysis. Similarly, in fly larvae, glycogen metabolism in the fat body plays a crucial role in the maintenance of circulating sugars under fasting conditions. One difference is that in *Drosophila*, glycogen from the fat body is converted to glucose, as well as trehalose, a form of nonreducing disaccharide primarily present in the insect hemolymph (65). The concentration of circulating trehalose concentration in third instar larvae is ~25 ug/ul, vs. ~0.1 ug/ul of glucose (66). In flies, excess energy from food, can result from feeding of flies on a high sugar diet, induces the expression of *glycogen synthase* (*glyS*), which promotes glycogen levels (10). Interestingly, unlike the action of glucagon in the mammalian liver, fasting-induced glycogen breakdown in the fly fat body is not regulated by AKH (Glucagon-like homolog in flies) (65). Instead, glycogen mobilization in the fat body is regulated by a decrease in sugar availability (65) and depends on glycogen phosphorylase (*GlyP*) (9), which increases in activity during larval development and remains high during pupal-adult development. In addition to *GlyP*, glycogen autophagy is also involved in glycogen breakdown in larval muscles (53). In adult flies, glycogen is stored mostly in the flight muscles, the fat body and oocytes. Whether oenocytes contribute to glycogen storage and mobilization is currently unclear and deserves further investigation.

Detoxification of toxic substances from the human body is mainly carried out by the liver. Detoxification are performed by phase I and phase II drug metabolizing enzymes (DMEs), as well as phase III transporters (54). Phase I DMEs consist of the cytochrome P450 (CYP) microsomal enzymes which are abundant in the liver, gastrointestinal tract, lung, and kidney. Phase II metabolizing and conjugating enzymes consist of superfamily of enzymes, including glutathione S-transferases (GSTs) (55–57). Interestingly, RiboTag analysis revealed that the microsomal GST *Mgstl* is highly enriched in *Drosophila* oenocytes (25). Further, transcriptome analysis in the yellow fever mosquito *Aedes aegypti* also showed that pupal oenocytes highly express cytochrome P450 genes (67), suggesting that oenocytes are responsible for detoxifications in insects. In addition to oenocytes, the fat body can also perform detoxification. For example, phenobarbital (present in insecticides) administration induced *Cyp6a2* expression in the *Drosophila* midgut and the pericuticular fat bodies (68). Also, biochemical analysis demonstrated that the fat body of the black swallowtail capillaries can metabolize linear furanocoumarins bergapten and xanthotoxin (69). Altogether, these data indicate that insect fat body and oenocytes can perform detoxification, analogous to the mammalian liver.

The liver, along with small intestines, kidneys and muscle are important organs for amino acid metabolism. The liver is the primary organ responsible for amino acids catabolism with the exception of branched amino acids. The amine group is separated and converted into urea, which is released into the blood and the remaining carbon group can be used for gluconeogenesis and ketogenesis (70). In honeybee (*Apis mellifera*) larvae, amino acids are synthesized into Hexamerins, which are storage proteins secreted by the fat body made of six polypeptide subunits. Hexamerins provide amino acids to other tissues during development and disappear during adulthood (71). In *Drosophila* larval storage proteins (Lsp) play a similar role (72). The fat body is also an important sensor for amino acids. Dietary amino acids are sensed by target of rapamycin (TOR)/regulatory associated protein of TOR (RAPTOR) in the *Drosophila* larval fat body, which can remotely control dILP release from the brain, thereby regulating systemic growth (73). In addition, an amino acid transporter, slimfast, can regulate TOR signaling in the fat body and regulates phosphatidyl-inositol 3-kinase (PI3K) signaling in peripheral tissues (74). Together, these results suggest that the insect fat body functions as a sensor and regulator that couples nutritional status to growth, through a humoral mechanism. However, whether insect fat body or oenocytes participate in amino acid catabolism and how they contribute to glycogen or carbohydrate metabolism remains unclear.

## Regulation of Fat Body and Oenocytes Metabolism Under Starvation

Perhaps the most distinct feature of oenocytes is their ability to accumulate lipids under starvation (6). Oenocytes are also important for the survival of the adult flies, as flies lacking oenocytes have increased sensitivity to starvation (13). Thus, oenocytes may process lipids or carbohydrates generated from the fat body to provide energy under starvation. In line with this, oenocyte ablation in larvae blocks fat body TAG depletion following starvation, suggesting a defect in lipid mobilization (6). Chatterjee et al. performed RNA-seq profiling under starvation conditions and showed that oenocytes containing carcasses had elevated gene expression in carbohydrate metabolism, the oxidation-reduction process, and amine metabolism. In contrast, the expression of genes involved in the defense response, chorion-containing eggshell formation, and proteolysis was increased in the fat body (13). Consistent with these observations, the classic catabolic and gluconeogenic genes *amylase proximal (amy-p)* and *phosphoenolpyruvate carboxykinase (pepck)* are induced in starved oenocytes. In addition, *lipophorin receptor 2 (lpr2)*, which is responsible for capturing lipids in the hemolymph, was also induced in oenocytes but not in the fat body (13). Altogether, these observations suggest that oenocytes and the fat body have different physiological response and that oenocytes might process lipids released from fat bodies under starvation.

## Roles of the Fat Body and Oenocytes in Immune Responses

Hepatocytes play an important role in controlling innate immunity via production of pattern-recognition receptors (PRRs) and pathogen associated molecular patterns (PAMPs) (75). As is the case for the mammalian liver, fly fat body and oenocytes express genes involved in immunity (25). Interestingly, the two innate immunity pathways (Toll and Imd) are differentially enriched in oenocytes vs. the fat body. Specifically, genes in the Imd pathway are enriched in oenocytes whereas Toll pathway genes are enriched in the fat body. The Toll pathway controls resistance to Gram-positive bacterial infections and fungal infections, and the Imd pathway controls resistance to Gram-negative bacterial infections (76). Downstream of Toll signaling are the NF-κB transcription factors: *dorsal* and *DIF*, whose immune-regulated functions are conserved in mammals (76). *Relish*, another NF-κB protein, is regulated by the Imd pathway and controls the expression of most of the *Drosophila* antimicrobial peptides (AMPs) (77). Further, many downstream effectors of *Relish* are highly enriched in oenocytes, including *DptA, DptB, CecC, Dro* and *MTK*. On the other hand, genes regulated by Toll signaling, including *Tl, PGRP-SA, GNBP3* and *modSP*, are preferentially expressed in the fat body (25).

## Nutrients and Tissues Regulating Lipid Metabolism in Oenocytes

Emerging evidence suggests that there is a close interaction between the fat body and the oenocytes in both larvae and adults. In larvae, the fat body consists of free-floating fat cells that are physically associated with the oenocytes (6, 78). In adults, fat body cells are tightly attached to the oenocytes (79). The fat body was the first tissue described to non-autonomously regulate oenocyte metabolism, as lipid accumulation in oenocytes depends on fat body nutritional sensors (6). Knockdown of *slimfast*, a fat body-specific amino acid transporter, causes lipid droplet accumulation in the oenocytes. Further, inhibition of TOR activity, following overexpression of TSC1 and TSC2 in the fat body, leads to a marked increase of steatosis in oenocytes. Similarly, inhibiting the phosphatidylinositol-3 kinase pathway through overexpression of PTEN also leads to severe steatosis in oenocytes, suggesting that oenocytes are regulated by the fat body nutritional status, either in response to fluctuating amino acid levels or through a TOR-dependent signaling peptide secreted by the fat body or by an intermediate tissue such as the intestine (Figure 1).

In addition to amino acids, oenocytes also respond to dietary lipids and fatty acids. When fed under high dietary lipids during larval stages, adult flies produce less pheromones (28), indicating that oenocytes VLCFA biosynthesis function is hindered in response to an increased level of circulating lipid (Figure 1). Further, overexpression of the Brummer (Bmm) lipase in the fat body induces steatosis in oenocytes under fed conditions. Conversely, overexpression of Lsd2 in the fat body under starved conditions reduces steatosis in oenocytes (6). Altogether, these results suggest that oenocytes develop steatosis when circulating levels of FFAs increase, which happens during starvation or under high fat diet treatment. This is consistent with what is observed in in vertebrates, in which either high fat diet or high levels of lipid uptake in hepatocytes all contributes to NAFLD pathogenesis (80–82).

In addition to nutritional signals, oenocytes are also regulated –by ligands received from other tissues under various conditions. The expression of *unpaired 2 (upd2)*, which encodes the first adipokine discovered in response to nutrients, is induced by dietary sugar and nutrients (83). Interestingly, oenocytes show abnormal steatosis in fed *upd2* mutants (83), suggesting that oenocytes might be regulated by fat-body derived upd2 in response to dietary nutrients. Unlike other tissues, such as the fat body, oenocytes exhibit increased levels of insulin activity when starved, as detected by the increased membrane localization of a PI3Kinase reporter (13, 84). Under starvation, the level of *dILP6* is elevated in the adult fat body (85), and overexpression of *dILP6* in the fat body can induce oenocyte insulin/IGF signaling (IIS) and steatosis in adult females (13). To the contrary, fat body-specific knockdown of dILP6 reduces the level of starvation-induced steatosis in oenocytes (13). Altogether, these data suggest that under starvation, the fat body signals to the oenocytes to increase lipid mobilization and carbohydrate metabolism via *dILP6* (Figure 1).

Oenocyte metabolism can also be regulated by a PDGF/VEGF-like ligand from the muscle. Ghosh et al. demonstrated that muscle-specific knockdown of *pvf1* leads to severe lipid accumulation in adipose tissue and oenocytes by increasing *de novo* lipogenesis (27). Single-nuclei RNA-sequencing (snRNA-seq) revealed that *PDGF- and VEGF-receptor related* (*Pvr*), which encodes the PVf1 receptor, is highly enriched in oenocytes. Further, this muscle-to-oenocyte signaling was found to inhibit rapid lipid expansion in newly eclosed flies. Altogether, these analyses suggest that Pvf1 acts as a myokine that suppresses oenocyte lipid synthesis (Figure 1). Interestingly, in vertebrates, VEGF-A and VEGF-B have been shown to be stored and released by muscles (86, 87). Furthermore, it has also been demonstrated that VEGF levels are lower in NAFLD patients than in healthy controls (88), and that a variety of myokines can reduce insulin resistance and fat accumulation in the liver (89). Thus, the *Drosophila* muscle-to-oenocyte model might be useful for discovery of new therapeutics for treating NAFLD.

## Crosstalk of Oenocytes With Other Tissues in Shaping Organismal Metabolism

Considering the role of oenocytes in metabolism, it is not surprising that many studies in which the function of oenocytes has been perturbed report observing defects in the metabolism of other tissues. For example, loss of oenocytes cytochrome P450, *Cyp4g1*, leads to an elevated oleic acid/stearic acid ratio, that is specific to TGs and not phospholipids, suggesting an altered fat body lipid composition and metabolism (6). Additionally, oenocyte-specific inhibition of TOR, following overexpression of *TSC1* and *TSC2* can induce lipogenesis and increase lipid storage in the fat body (27). Further, oenocyte ablation or knockdown of genes encoding VLCFA metabolizing enzymes in larvae leads to severe tracheal defects and altered spiracle lipid metabolism (31). In addition, in a genome-wide RNAi screen in *Drosophila* for obesity-causing genes, many genes were identified that regulate whole-body fat content in an oenocyte-dependent manner (90). Interesting targets included inflammation-related genes and genes regulating ubiquitination, including TNF-receptor-associated factor 4 (*Traf4*), the interleukin enhancer-binding factor (*ILF2)*, and the ubiquitin-conjugating enzyme *(UBE2N)* (90). These data suggest a role for immune regulatory networks and the ubiquitination in regulating fat storage in oenocytes. Oenocytes can also regulate vitellogenesis during oogenesis, as oenocyte-specific knock down of the *seven up (svp)*, which encodes a nuclear receptor, can increase vitellogenic follicle egg chamber death (30). Moreover, *svp* is a known oenocyte-specific modulator of cuticle lipids, raising the possibility that female oenocytes regulate vitellogenic follicles through the production of hydrocarbons (Figure 1).

## Conclusions

Major advances have been made in recent years in understanding the role of insect oenocytes in metabolism and physiology, and many fascinating research areas remain unexplored. One largely ignored yet important area is to delineate the differences and similarities between oenocyte function in *Drosophila* larvae and adults. Understanding such differences will be important for choosing the most appropriate stage to model a specific human disease. Another area of interest is the synthesis of VLCFAs and their derivatives in oenocytes, as this in turn affects not only hydrocarbon synthesis but also lipid metabolism in trachea, fat body and oocytes. Such non-autonomous regulation remains largely obscure and may be mediated through metabolites or signaling pathway activities. Finally, it is highly likely that additional oenocyte functions and crosstalk with additional tissues remain to be identified. For example, the role of oenocytes in immunity and bacterial defense, as suggested by the expression of IMD signaling pathway genes, need to be clarified. The role of oenocytes in drug detoxification as well as amino acids metabolism remains elusive. In addition, whether oenocyte metabolic functions regulate aspects of fly behavior, other than mating, remains to be explored.

## Author Contributions

KH drafted and revised the manuscript. YL and NP helped to draft and revise the manuscript. All authors contributed to the article and approved the submitted version.

## Conflict of Interest

The authors declare that the research was conducted in the absence of any commercial or financial relationships that could be construed as a potential conflict of interest.

## Publisher's Note

All claims expressed in this article are solely those of the authors and do not necessarily represent those of their affiliated organizations, or those of the publisher, the editors and the reviewers. Any product that may be evaluated in this article, or claim that may be made by its manufacturer, is not guaranteed or endorsed by the publisher.
